# Wild-Type Transthyretin Amyloid Cardiomyopathy With Bone Marrow Involvement: Clinical Implications of Less Common Sites of Amyloid Deposition

**DOI:** 10.7759/cureus.92835

**Published:** 2025-09-21

**Authors:** Yusuke Kashiwagi, Kazuhito Suzuki, Tsuneaki Yoshinaga, Mitsuto Sato, Kae Kawachi, Kazuo Ogawa, Moroe Beppu, Michifumi Tokuda

**Affiliations:** 1 Cardiology, The Jikei University School of Medicine, Tokyo, JPN; 2 Clinical Oncology/Hematology, The Jikei University School of Medicine, Tokyo, JPN; 3 Neurology, Shinshu University School of Medicine, Matsumoto, JPN; 4 Pathology, The Jikei University School of Medicine, Tokyo, JPN; 5 Orthopaedic Surgery, St. Marianna University School of Medicine, Kawasaki, JPN

**Keywords:** bone marrow, carpal tunnel syndrome, heart failure, technetium-99m pyrophosphate, transthyretin amyloid cardiomyopathy

## Abstract

Transthyretin amyloidosis (ATTR) is a progressive disease characterized by tissue deposition of transthyretin (TTR)-derived amyloid fibrils, commonly involving the heart, joints, and ligaments. However, deposition in less common sites, such as the bone marrow, has also been reported, but its clinical significance remains unclear. An 86-year-old man presented with mild heart failure. Amyloid was initially detected in synovial tissue during carpal tunnel release surgery, which prompted technetium-99m pyrophosphate (99mTc-PYP) scintigraphy, and an endomyocardial biopsy confirmed wild-type ATTR cardiomyopathy (CM). Due to an elevated serum-free light chain ratio, a bone marrow biopsy was performed to exclude immunoglobulin light chain (AL) amyloidosis or myeloma, revealing amyloid deposits positive for TTR and negative for both anti-κ and anti-λ antibodies, indicating ATTR-type deposition. A TTR stabilizer was initiated shortly after diagnosis. Although biomarker levels, such as B-type natriuretic peptide and troponin I, and echocardiographic findings were consistent with mild disease at presentation, both gradually worsened over the subsequent year, in parallel with increasing heart failure symptoms. This case highlights that TTR amyloid deposition in a less common site (e.g., the bone marrow), even when the initial clinical findings are mild, may reflect a more advanced stage of wild-type ATTR-CM.

## Introduction

Transthyretin amyloid cardiomyopathy (ATTR-CM) is a progressive and life-threatening condition characterized by the deposition of transthyretin (TTR)-derived amyloid fibrils in the myocardium. The emergence of disease-specific therapies, such as agents that stabilize TTR tetramers, has led to improved outcomes in patients with ATTR-CM [1-3], and in particular, tafamidis, a TTR stabilizer, has been reported to be more effective in the relatively early stages of the disease [1], thus highlighting the importance of early diagnosis and appropriate and timely initiation of treatment. Additionally, identifying reliable indicators that can predict disease progression and prognosis is becoming increasingly important for optimizing patient management.

In amyloidosis, the organs involved in amyloid deposition and the resulting organ dysfunction are critical factors in assessing disease progression and the prognosis. AL amyloidosis is a systemic disease in which amyloid deposits affect multiple organs, including the heart, kidneys, gastrointestinal tract, and peripheral nerves [4,5]. In contrast, ATTR amyloidosis, especially wild-type, is commonly deposited in a limited set of organs (joints, ligaments, and heart) [6], and the significance of deposition in less common sites, such as the bone marrow, has not been fully elucidated.

We herein report a case of wild-type ATTR-CM in which histopathological analysis unexpectedly revealed TTR-derived amyloid deposits in the bone marrow, which is a less common site of involvement in transthyretin amyloidosis. This case suggests that amyloid deposition in less common sites (e.g., the bone marrow) may reflect a more advanced stage of wild-type ATTR-CM and could represent a potential precursor to subsequent clinical deterioration.

## Case presentation

**Figure 1:**
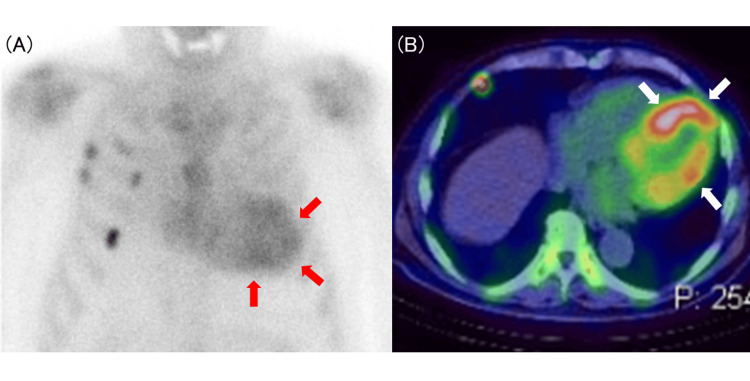
Nuclear imaging with technetium-99 m pyrophosphate (99mTc-PYP) scintigraphy. (A) 99mTc-PYP scintigraphy showed significant myocardial accumulation (Perugini grade 3) with a myocardial-to-lung ratio of 1.68 (planner anterior view). (B) Trans-axial view of a single-photon emission computed tomography (SPECT) image. Arrows indicate 99mTc-PYP uptake in the myocardium, with red arrows in (A) and white arrows in (B).

An 86-year-old man presented with mild symptoms corresponding to the lower end of New York Heart Association (NYHA) functional class II, including slight exertional dyspnea of one-year duration and mild lower extremity edema. On a physical examination, his blood pressure was 133/70 mmHg, and his heart rate was 65 beats per minute. Cardiac auscultation revealed normal heart sounds without murmurs. His medical history included hypertension, chronic kidney disease (CKD), and right knee arthroplasty performed six years earlier. Approximately one month prior, he had undergone surgery for left carpal tunnel syndrome, during which amyloid deposits were found in the synovial tissue of the surgical specimen. Technetium-99m pyrophosphate (99mTc-PYP) scintigraphy revealed significant myocardial accumulation (Perugini grade 3) with a heart-to-contralateral lung (H/CL) ratio of 1.68 (Figures 1A, 1B).

**Figure 2:**
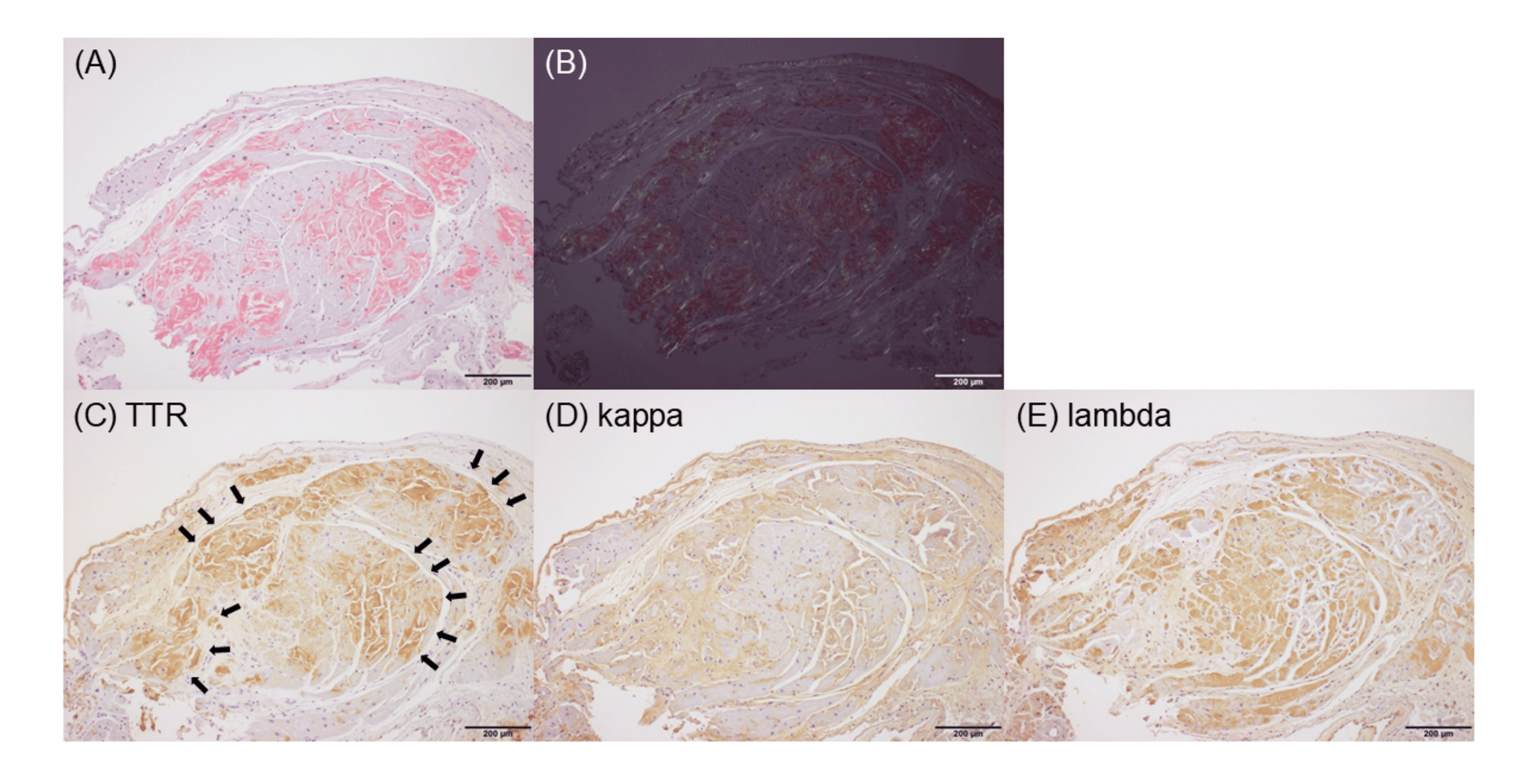
Histological findings of the endomyocardial biopsy specimen from the left ventricle. (A) Amyloid deposits, appearing orange on direct fast scarlet (DFS) staining, were observed between the myocardial fibers. (B) DFS staining under polarized light. (C-E) Immunohistochemical staining of separate specimens for transthyretin (TTR) (C), kappa (κ) light chains (D), and lambda (λ) light chains (E). Consistent with the DFS-positive area, positive staining was observed in the TTR (black arrows), while κ- and λ-light chains were negative (some nonspecific staining was observed in κ- and λ-). Scale bar: 200 µm.

Right heart catheterization revealed a pulmonary artery wedge pressure of 22 mmHg with a cardiac index of 3.26 L/min/m^2^ (thermodilution) and 3.67 L/min/m^2^ (Fick). An endomyocardial biopsy revealed amyloid deposits that appeared orange with direct fast scarlet (DFS) staining and yellow-green under polarized light between the myocardial fibers (Figures 2A, 2B). Immunohistochemical analysis showed that the ATTR was positive, consistent with DFS-positive sites, and kappa (κ) and lambda (λ) light chains were negative (Figures 2C-2E). Subsequent genetic testing confirmed the diagnosis of the wild-type ATTR.

In addition, the serum-free light chain ratio (κ/λ ratio) increased to 12.01, with κ light chains at 275 mg/L and λ light chains at 22.9 mg/L. Since multiple myeloma was suspected, a bone marrow biopsy was performed, which revealed both amyloid deposits and proliferation of plasma cells showing κ light chain restriction by immunohistochemistry (percentage of plasma cells: 9.0%, which did not meet the diagnostic criteria for myeloma). The diagnosis based on these findings was considered monoclonal gammopathy of undetermined significance.

**Figure 3:**
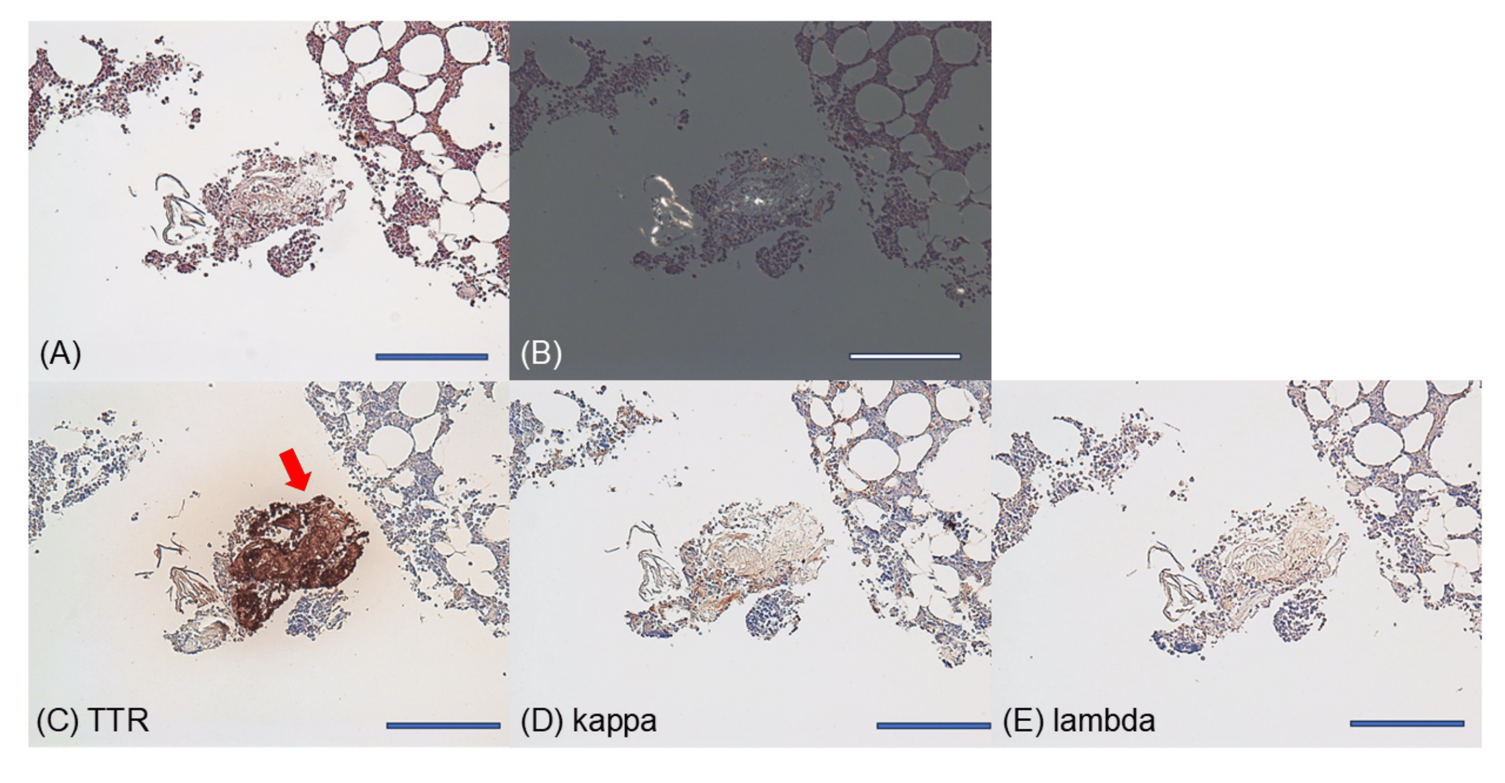
Histological findings of the bone marrow biopsy specimens. (A) Congo red staining. (B) Congo red staining under polarized light. (C-E) Immunohistochemical staining of separate specimens for transthyretin (TTR) (C), kappa (κ) light chains (D), and lambda (λ) light chains (E). Positive staining was observed for TTR (red arrow), whereas κ- and λ-light chains were negative. Scale bars: 200 μm.

Histologically, the marrow was composed of hematopoietic tissue and bony trabeculae, and amyloid deposits were observed within the vascular structures of hematopoietic areas using Congo red staining, showing apple-green birefringence under polarized light (Figures 3A, 3B). Immunohistochemical staining was positive for anti-TTR antibodies and negative for both anti-κ and anti-λ antibodies (Figures 3C-3E). These results indicated a diagnosis of ATTR cardiac amyloidosis accompanied by ATTR amyloid deposition in the bone marrow, a non-prevalent site. During the clinical course, the patient showed no macroglossia or gastrointestinal symptoms such as diarrhea or proteinuria, supporting the exclusion of AL amyloidosis.

After the diagnosis of ATTR-CM, treatment with tafamidis (61 mg orally once daily) was initiated. However, the patient’s heart failure gradually progressed over the subsequent year. As shown in Table 1, at the time of diagnosis, the symptoms were classified as NYHA class II but later deteriorated to class III. B‑type natriuretic peptide (BNP) levels increased from 114 pg/mL at baseline to 349 pg/mL after one year. Similarly, troponin I levels increased from 111 to 167 pg/mL over the same period. Echocardiography revealed that several parameters, including systolic and diastolic function, steadily worsened over the course of the year. Initially, the patient was managed with a low dose of diuretics (torasemide 2 mg/day); however, after one year, the dose was escalated to furosemide 20 mg/day owing to worsening congestion (Table 2).

**Table 1:** A comparison of heart failure-related parameters. NYHA, New York Heart Association classification; BNP, B‑type natriuretic peptide; LVEF, left ventricular ejection fraction; IVS, interventricular septal thickness; PW, posterior wall thickness; E/e', the ratio of peak early diastolic transmitral flow velocity (E) to early diastolic mitral annular velocity (e'); TR, tricuspid regurgitation; GLS, global longitudinal strain. Normal reference ranges: BNP, 0–18.4 pg/mL; Troponin I, 0–23 pg/mL.

	At the initial assessment	At one-year follow-up
NYHA	Ⅱ	Ⅲ
Body weight (kg)	72	73.2
BNP (pg/mL)	114	349
Troponin I (pg/mL)	111	167
Echocardiographic data		
Granular sparkling	Unclear	Present
LVEF (%)	58.8	53.6
IVS (mm)	13.6	14.4
PW (mm)	12.3	13.6
E/E`	11.9	16.5
TR pressure gradient (mmHg)	32.65	55.03
GLS (%)	Not available	15.3

**Table 2:** Clinical timeline of the patient. NYHA, New York Heart Association classification; 99mTc-PYP, technetium-99m pyrophosphate; H/CL, heart-to-contralateral lung; ATTR-CM, transthyretin amyloid cardiomyopathy; BNP, B‑type natriuretic peptide.

Time	Event
–12 months	Onset of mild exertional dyspnea
–1 month	Carpal tunnel surgery; amyloid deposits found in the synovial tissue
0 month	Initial presentation (NYHA II)
+1 month	99mTc-PYP scintigraphy performed: Perugini grade 3, H/CL ratio 1.68
+2 months	Endomyocardial biopsy performed (ATTR positive)
+2 months	Bone marrow biopsy: TTR amyloid deposition
+3 months	Genetic testing confirmed wild-type ATTR-CM
+3 months	Tafamidis (61 mg/day) initiated
+12 months	Progression to NYHA III; BNP and troponin I increased; worsening cardiac function; diuretics escalated

## Discussion

In the present case, TTR deposition was observed in the bone marrow, which is an uncommon site for wild-type transthyretin amyloidosis (ATTRwt). At the time of the diagnosis, the patient presented with only mild heart failure symptoms corresponding to NYHA class II, and both biomarker levels and echocardiographic findings were consistent with those of the early stages of the disease. Nevertheless, despite the initiation of TTR tetramer stabilizer, the patient’s heart failure gradually worsened over the course of the year, in parallel with a progressive increase in cardiac biomarkers. These findings suggest that amyloid deposition in less common sites, such as the bone marrow, is potentially relevant for assessing disease severity and progression.

Previous studies have proposed prognostic staging systems for wild-type ATTR amyloidosis based on N-terminal pro-BNP (NT-proBNP), eGFR, and troponin T [7,8]. Although the prognosis of ATTRwt cardiac amyloidosis is generally better than that of AL cardiac amyloidosis [9], a previous study suggested that patients with ATTR deposition in the bone marrow and cardiac involvement have inferior survival rates in comparison to those with AL amyloidosis and cardiac involvement [10]. Thus, the presence of TTR deposition in bone marrow may indicate a potentially poor prognosis in patients with ATTR-CM. In other words, deposition of transthyretin amyloid in less common sites, such as the bone marrow, in addition to its more common locations, may reflect disease progression and could serve as a marker for an advanced disease stage. We hypothesize that amyloid involvement of less common sites, such as the bone marrow, in ATTRwt amyloidosis may represent a potential indicator of heightened systemic amyloid burden, although the precise mechanism underlying TTR amyloid deposition in the bone marrow remains unclear. If amyloid deposition in less common sites reflects subclinical progression before the worsening of heart failure symptoms, it could serve as a complementary indicator to conventional markers.

In this case, due to the patient’s elevated serum free light chain ratio, a bone marrow biopsy was performed to exclude AL amyloidosis and multiple myeloma. Histopathological evaluation revealed amyloid deposits that stained positive for TTR, but negative for κ- and λ-light chains, confirming ATTR-type deposition. In AL amyloidosis, the frequency of amyloid deposition in bone marrow biopsy specimens is reported to be approximately 60% [11,12]. In contrast, a recent study from Japan demonstrated that amyloid deposition in the bone marrow was observed in only 2.8% of ATTRwt cases [6]. This low frequency may be explained by the fact that, in general, bone marrow biopsies are rarely performed in ATTRwt patients, unless an abnormal κ/λ light chain ratio or other clinical indications are present. A report from the Mayo Clinic indicated that bone marrow biopsies were performed in 64% of ATTRwt amyloidosis cases, and among those, amyloid deposition was positive in approximately 30% [13]. Considering this, amyloid deposition in the bone marrow may be relatively more common in ATTR amyloidosis than previously thought, and a bone marrow biopsy in patients with indications, such as an elevated serum free light chain ratio, may provide an additional indicator for assessing disease severity.

## Conclusions

This case highlights the fact that TTR deposition in the bone marrow, even when initial clinical findings are mild, may indicate a more advanced stage of wild-type ATTR-CM. Such a less common deposition may reflect subclinical disease severity and, when combined with established prognostic markers, serve as an additional marker for risk stratification and help inform earlier initiation of disease-specific therapies (e.g., TTR tetramer stabilizers and TTR gene silencers). Furthermore, it is possible that such deposition in the bone marrow may be observed more frequently than previously thought when biopsies are performed proactively. Given that this is a single-case report, further studies are needed to determine whether or not deposition in less common sites, such as the bone marrow, has prognostic significance.
